# Correction to: Glucose control in diabetes during home confinement for the first pandemic wave of COVID‑19: a meta‑analysis of observational studies

**DOI:** 10.1007/s00592-021-01780-0

**Published:** 2021-08-21

**Authors:** Giovanni Antonio Silverii, Chiara Delli Poggi, Ilaria Dicembrini, Matteo Monami, Edoardo Mannucci

**Affiliations:** Diabetes Unit, Experimental and Clinical Biomedical, Sciences “Mario Serio” Department, AOU Careggi Hospital, University of Florence, Largo Brambilla 3, 50134 Florence, Italy

## Correction to: Acta Diabetologica 10.1007/s00592-021-01754-2

Authors would like to correct the given names and family names of authors. The correct list of the authors is Giovanni Antonio Silverii, Chiara Delli Poggi, Ilaria Dicembrini, Matteo Monami, Edoardo Mannucci.

The original article has been corrected.

